# Supplementary material for: Patterns of Intron Gain and Loss in Fungi
Source: PLoS Biol. 2004 Nov 30;2(12):e422. doi: 10.1371/journal.pbio.0020422 (PMC532390; doi:10.1371/journal.pbio.0020422)
Supplement: Table S1 — Also available at http://genes.mit.edu/NielsenEtAl/. (4.3 MB ZIP). [file pbio.0020422.st001.zip › NielsenEtAl/html/201.html]

AN7031.1.NCU06594.1.MG01703.1.FG05262.1


```
 CLUSTAL W (1.82) Multiple Sequence Alignments - Introns Inserted


Sequence 1: NCU06594.1	517 aa
Sequence 2: FG05262.1	509 aa
Sequence 3: MG01703.1	508 aa
Sequence 4: AN7031.1	453 aa
Alignment Length: 526 aa
Number Identitical Residues: 179 aa
Alignment Score (without introns) 9674


MG01703.1 	-MPNTSWSQAAGIPHAYASYS---~PV1R~QSFGPVASAQ--HSYS--PSQGTSYPSHPG
NCU06594.1	-MQWSSAQPPPPGTQPPAAYPYSV1QV~R~QTFGQPYSAAPPASYPPPPAYGPLPINPPP
FG05262.1 	MMPTWSAQQAPSVTQQTVPASYAY~PT~N1PAFIPVAVRQGFNQYGVPPPYAGYAPPPPP
AN7031.1  	-MSSGIPPWRASASATATTAVYPS~HA1T~PAYIPVQARR-----------SLTTPASTS
          	 *        .  .   ..   .   .    ::                  .      . 

MG01703.1 	ATSTTTETAAQPTEQS---------KKIDWPLSVRQYVARSFLPDNMDHTVDRTEMEAKL
NCU06594.1	ATPLHTNPAAPLTAATPTTTQEPAKKKIDWPESVRNYVQRSFQTSNLDPSVTRAEMEAKL
FG05262.1 	VQPQMS-SASPVNGVT--ATPEQAKSKVDWPESVRSYVQRSFLPQYDEPTVPRAEIEAKL
AN7031.1  	ATPAPQPKTTAADAGN--------RKRVEWPEPVRKYVQRCFAPENQISGVSRQEMEAKL
          	. .     ::     .         .:::** .**.** *.* ..     * * *:****

MG01703.1 	KETISNAHQTGVMYTTDWDKYPLPQELIKQERLASLMSTTVALPNTTWNVATPTQKKRKS
NCU06594.1	KETISYANDQGHMYTVNWEKMPLPQEMIKEERARARA-LQQASHFSALSISPSSSKKRKS
FG05262.1 	KDTIGTAKANGTLYTLDWDNMPLPQALVKAERD--------ASTKQSFQV-PLNSKKRKS
AN7031.1  	RSVITEAAQSDVLDKINWEAQPLPQVMVQ---------------NDKLRILTNPEASTWG
          	:..*  *   . : . :*:  **** :::                    : .  . .  .

MG01703.1 	SDMSSEGLPGATAPWQSAKSNPLESRVSYQTTDKRPAPGASSNKAAMSKFQKQLEKRQRR
NCU06594.1	TDFS----EGAAPPWR-NGQNRLEDRITFNA-DKRPAPVEEAQSKSQSKFQKQLEKRQKR
FG05262.1 	TDFACN--DNSQPPWRTNSRSSLEDRISFSS-PEKRASMDEPLPKS-SKFQKEANKRKRR
AN7031.1  	TPFAAQ------------------------S-PKRTASDDASRKRKSAEYQTESN-----
          	: ::..                        :  :: *.   .     :::*.: :     

MG01703.1 	FDGGYKSTYRSPSPPPSDGPIVGTCQTLEKRYLRLTGPPVISQV~RPEPILHQTLDLLKK
NCU06594.1	FDGGYKSTYREPTPPPSDGPVVGTCETLEKRYLRLTAPPVPSVV~RPERVLRQTLELLKR
FG05262.1 	FENEYKSLRSPSPPPPSSGPIVGTCEVLEKKYLRLTAPPVPSKV~RPESILRQTLDLLKK
AN7031.1  	--------ACPPWRQTNKSNVFEDRVTYSPTDKRQRIESKNSNI1RPLPVLMKTLDLLKK
          	           .   .... :.    . .    *    .  * : **  :* :**:***:

MG01703.1 	KWRKESNYSYICDQFKSLRQDLTVQHIKNEFTVSVYEIHARIALEKGDLGEYNQCQTQLR
NCU06594.1	KWKKEQNYSYICDQFKSMRQDLTVQRIKNEFTVEVYEIHARIALEKGDLGEYNQCQTQLK
FG05262.1 	KWKRESNYSYICDQFKSMRQDLTVQHIKNDFTVSVYEIHARIALEKGDIGEYNQCQTQLR
AN7031.1  	KWKKDNNYTYICDQFKSLRQDLTVQHIRNEFTVSVYEIHARIALEKGDLGEYNQCQTQLR
          	**:::.**:********:*******:*:*:***.**************:**********:

MG01703.1 	ALYQLGLKGNPLEFKAYRILYFIHTANRSALNDAMADLTPAEKEERPLKHALNVRSALAL
NCU06594.1	ALYKMGIKGKSIEFKAYRILYFIHTANRTALNDVLADLTAAEKEEEAIKHALDVRSALAL
FG05262.1 	SLYGMGLKGNPIEFKAYRILYFIHTANRTGLNDTLADLTTAEKEEKAIKHALNVRSSLAL
AN7031.1  	ALYAQQLGGHPTEFKAYRILYFIHTRNWTAMNDALADLIPADKRSAAVKHALDVRSALAL
          	:**   : *:. ************* * :.:**.:*** .*:*.. .:****:***:***

MG01703.1 	GNYHRFFQLYLDTPNMGAYLMDMFVGRERLAALCNICRA2YKPDVNLRFVTEELGFESDA
NCU06594.1	GNYHRFFRLYNDTPNMGAYLMDMFVGRERLAALCNICKA2YKPDVPLRFVTEELYFESDA
FG05262.1 	GNYHRFFQLYLDTPNMGAYLMDMFVVRERLAALCNICKA2YKPDVKLRFITEELGFESDA
AN7031.1  	GNYHRFFQLYLATPNMGAYLMDMFVDRERLSALSAICKA2YKPDVKIRFITEELGFESDE
          	*******:**  ************* ****:**. **:* ***** :**:**** **** 

MG01703.1 	EAVQFIIDHNGQDLLEERPDYIAFATG-KAGQRFEALRAEAFRRVDIKGQI
NCU06594.1	DAAQFILDYQGQDLLEDREGTVMFLTG-KAKELFEGPRKAAFSRVDIKGQI
FG05262.1 	DAAQFVIDHQGQHLLEDRTDYIAFLTG-KAGPLFEAARSTAFQKVDIKGQI
AN7031.1  	QSARFILDHSSEDMLLEKDGSVRLLTGGKAAQLFEAAKADAHRVIDIKGQI
          	::.:*::*:..:.:* :: . : : **.**   **. :  *.  :******
```
